# Alveolar Bone Loss in a Ligature-Induced Periodontitis Model in Rat Using Different Ligature Sizes

**DOI:** 10.1055/s-0044-1779426

**Published:** 2024-03-05

**Authors:** Warintorn Wichienrat, Theeraphat Surisaeng, Noppadol Sa-Ard-Iam, Theerapat Chanamuangkon, Rangsini Mahanonda, Wichaya Wisitrasameewong

**Affiliations:** 1 Department of Periodontology, Faculty of Dentistry, Chulalongkorn University, Bangkok, Thailand; 2 Center of Excellence in Periodontal Disease and Dental Implant, Faculty of Dentistry, Chulalongkorn University, Bangkok, Thailand; 3 Immunology Research Center, Faculty of Dentistry, Chulalongkorn University, Bangkok, Thailand; 4 Biomaterial Testing Center, Faculty of Dentistry, Chulalongkorn University, Bangkok, Thailand

**Keywords:** ligature-induced periodontitis model, alveolar bone loss, rodent

## Abstract

**Objectives** Ligature-induced periodontitis model has been widely used as a preclinical stage for investigating new treatment modalities. However, the effect of different ligature sizes on alveolar bone loss has never been studied. Therefore, we examined alveolar bone loss in this rat model using different sizes of silk ligatures, as well as healing after ligature removal.

**Materials and Methods** Left maxillary second molars of Sprague-Dawley rats were ligated with 3-0, 4-0, or 5-0 silk ligatures ( *n* = 4–5/group) for 14 days before harvested maxillae and gingival tissues. For subsequent experiment, animals were ligated for 14 days using the ligature size that induced the most alveolar bone loss before ligature removal and sacrificed at 0, 7 and 14 days ( *n* = 5–6/group). All maxillae and gingival tissues were harvested to evaluate alveolar bone level, tumor necrosis factor-α (TNF-α), and interleukin-1β (IL-1β) levels.

**Statistical Analysis** Data was analyzed using SPSS Statistics 23.0 software (SPSS Inc., Chicago, Illinois, United States). Data from all experiments were tested for normality using Shapiro–Wilk test. Data between ligatured and nonligatured teeth were compared using Student's *t* -test or Wilcoxon signed-rank test. Differences among different ligature sizes were analyzed by analysis of variance followed by multiple comparisons with post-hoc test. A *p-* value less than 0.05 was considered statistically significant.

**Results** The alveolar bone loss of ligated teeth was substantially higher than that of control after 14 days of ligation. While 3-0 and 4-0 resulted in significantly greater bone loss than 5-0 silk, the 3-0 group had the lowest rate of ligature loss. Therefore, alveolar bone healing postligature removal was investigated further using 3-0 silk. The results showed no significant bone level change at 2 weeks after ligature removal. In term of IL-1β and TNF-α levels, there was no statistically significant difference in IL-1β level between groups at any time point, while TNF-α was undetectable.

**Conclusion** These data showed that 3-0 silk was the most effective ligature size in promoting alveolar bone loss comparing with 4-0 and 5-0 silk. During the 2-week period following ligature removal, spontaneous bone healing was not observed.

## Introduction

Animal models are beneficial and have been widely used as a preclinical stage to test the new regenerative biomaterials and treatment techniques. Rodents are commonly used in various disease models, including periodontitis. The major advantages of rodent model compared to other animals are its availability, relatively lower cost, ease of handling, ability to perform specific study model owing to various genetic and microbial profile available, and a rapid disease development by various methods.

In an aspect of periodontal diseases, rodent shares some similarities with human, including periodontal structure, molar tooth anatomy, microbial plaque formation, and development of periodontal lesions in terms of bacteria-induced and RANKL-dependent periodontal bone loss. 1 2 3 4 Therefore, various approaches have been applied to induce the experimental periodontitis in rodents, for example, oral gavage or oral inoculation with human periodontal pathogens, such as *Porphyromonas gingivalis* ( *P. gingivalis* ), *Fusobacterium nucleatum* ( *F. nucleatum* ), *Prevotella intermedia (P. intermedia),* and *Aggregatibacter actinomycetemcomitans (A. actinomycetemcomitans)* . 3 4 5 6 Moreover, experimental periodontitis model can also be developed using a silk ligature to facilitate plaque accumulation around teeth, leading to inflammation and, eventually, alveolar bone loss. 7 8 9 10 11 Other than the use of pathogens, periodontal defects can be created surgically, such as intrabony defect and buccal fenestration. This technique could create periodontal defects at various sites, such as at mesial aspect of maxillary first molars, 1 12 mesial root of mandibular first molar, 13 14 buccal/ distal root of mandibular first molar, and mesial root of mandibular second molar. 15

However, in experimental periodontitis rat model, surgically created periodontal defects are technique sensitive with high mortality rate observed. In addition, there is very little or no effect of bacterial plaque infection in causing the lesion; thus, this model may not reflect the natural pathogenesis of periodontitis as occurred in human. 16 For the bacteria-induced model, de Molon et al evaluated two types of experimental periodontitis models in mice: oral gavage and ligature-induced. The results demonstrated that induction of periodontitis using oral gavage with *P. gingivalis* and *F. nucleatum* can cause the colonization of *P. gingivalis* after 45 days and 60 days of disease induction. However, oral gavage was less effective in providing plaque accumulation, induction of periodontal inflammation and alveolar bone loss compared to ligature method. 5 In addition, oral gavage took months to develop the disease, whereas ligature method could develop disease within a few weeks. Besides, the pathogenesis of periodontal disease in ligature model is more similar to human periodontitis. 8 11 17 18 19 Ligatures could induce rat microbiome dysbiosis by promoting plaque accumulation, which increase microbiome complexity and biomass. Even though the organisms found in experimental periodontitis rat are not at all related to human organisms, dysbiosis does occur, leading to inflammation and, subsequently, periodontal destruction. 18 Despite the aforementioned advantages, the limitations of the rodent model are its enhanced alveolar bone remodeling and slow passive tooth eruption throughout life for compensating of rapid occlusal wear. 1 20 Furthermore, wound healing process in rodent occurs more rapidly compared to human. 21 These may affect the amount of true inflammatory bone loss and negatively affect the outcome of experimental study aiming to test the regenerative ability of biomaterials or treatment modalities. To the best of our knowledge, the effect of different ligature sizes on alveolar bone loss and bone healing after ligature removal have never been investigated.

Therefore, in our study, we proposed to examine the alveolar bone loss in ligature-induced experimental periodontitis using different sizes of silk ligature. The alveolar bone healing without intervention following ligature removal would also be studied.

## Materials and Methods

### Animals

All animal experimental procedures were ethically reviewed and approved by the Chulalongkorn University-Animal Care and Use Committee and Faculty of Tropical Medicine-Institute Animal Care and Use Committee at Mahidol University, and Animal Research: Reporting of In Vivo Experiments (ARRIVE) guideline were followed. Five to six animals per group were required based on the calculation according to a statistical power of 80%, α = 0.01 and 20% compensation for animal loss.

Thirty-three Sprague-Dawley male rats at the age of 6 weeks were purchased from Nomura Siam International (Bangkok, Thailand) and adopted in individually ventilated cage with 12-hour light/dark cycle for a week before beginning of the experiment at the Laboratory Animal Science Unit of Faculty of Tropical Medicine, Mahidol University.

### Ligature Placement with Different Sizes of Silk Ligature

Fifteen rats were randomly assigned into one of three groups: 3-0, 4-0, and 5-0 silk ligature (5 rats/group). A ligature was ligated around the left maxillary second molars and tied firmly on the buccal using a triple-knot with slightly modified from Abe and Hajishengallis, 8 while the right maxillary second molars were left nonligated as a control site.

The ligatures were checked in all animals at day 7 postligation. Animals with ligature loss would be excluded from the study. Fourteen days postligation, all animals were sacrificed. Maxilla and gingival tissue were harvested for further analysis.

### Ligature Placement and Removal

The ligature size that caused maximum bone loss from previous experiment was further investigated in this experiment by ligating in 18 rats as described previously and was randomized into three groups ( *n* = 6/group). All animals were anesthetized, and the ligatures were removed at 14 days. Animals in group 1 were then immediately sacrificed and the other animals in group 2 and 3 were sacrificed at 7 and 14 days, respectively.

After sacrificed and dissected the maxilla, one sample from each time point was used for histologic evaluation and the remaining samples were used to evaluate alveolar bone and pro-inflammatory cytokines levels.

### Evaluation of Alveolar Bone Level

Maxillae were dissected and gingival tissues were removed. Maxillae were immersed in 5% sodium hypochlorite for 5 days, washed in running water and dried. Alveolar bone level was measured under stereomicroscope (Olympus SZ61; Olympus Corporation, Tokyo, Japan) and the images were analyzed using ImageJ 1.52a software program (National Institutes of Health, Bethesda, Maryland, United States). Some specimens were further analyzed using micro-computed tomography system (Micro-CT µ35 scanco; SCANCO medical, Brüttisellen, Switzerland) and three-dimensional (3D) digital images of the maxilla were generated using the 3D reconstruction software. “Alveolar bone loss” was calculated as mean values of a distance between cementoenamel junction (CEJ) to the alveolar bone crest (ABC), which was measured at buccal (5 sites) and palatal (5 sites) area at distobuccal/palatal line angle of maxillary first molar, mesiobuccal/palatal line angle, mid-buccal/palatal, and distobuccal/palatal line angle of second molar and mesiobuccal/palatal line angle of third molar. “Alveolar bone healing” was calculated by the change in alveolar bone loss from day 0 to day 7 and day 14 after ligature removal. All measurements were performed by one examiner who was blinded to the specimen's group.

### Histologic Evaluation of Alveolar Bone Level

Maxillae were fixed in 10% formaldehyde overnight. The specimens were decalcified in 10% ethylenediaminetetraacetic acid solution at 4°C for 2 weeks before being embedded in paraffin. The specimens were serially sectioned at 7 µm thickness in the mesiodistal direction and stained with hematoxylin and eosin. The sections were examined under a light microscope.

### Measurement of Proinflammatory Cytokines in Gingival Tissue

Gingival tissues were harvested and homogenized in 1 mL of phosphate-buffered saline with protease inhibitor cocktail (Sigma, St. Louis, Missouri, United States). The homogenates were centrifuged at 16,000 rpm for 15 minutes at 4°C. The amount of total proteins was measured by BCA protein assay kit (PierceTM BCA Protein Assay; Thermo Scientific, Co., Ltd., Rockford, IL United States). The levels of IL-1β and TNF-α were measured using enzyme-linked immunosorbent assay (ELISA) kits (Quantikine ELISA; R&D System, Inc., Minneapolis, Minnesota, United States).

### Statistical Analysis

Data was analyzed using SPSS Statistics 23.0 software (SPSS Inc., Chicago, Illinois, United States). Data from all experiments were tested for normality using Shapiro–Wilk test. Data between ligatured and nonligatured teeth were compared using Student's *t* -test or Wilcoxon signed-rank test. Differences among different ligature sizes were analyzed by analysis of variance followed by multiple comparisons with post-hoc test. A *p-* value less than 0.05 was considered statistically significant.

## Results

The ligature was checked 1 week after being placed around the left maxillary second molar. The incidence of ligature loss varied according to ligature size. The percentage of ligature loss was highest in the size 5-0 group (53.8%), followed by the 4-0 (37.5%) and 3-0 (28.6%) groups, respectively. The animals were excluded from the study.

### Alveolar Bone Loss and Proinflammatory Cytokine Levels Comparing between Different Ligature Sizes

**Fig. 1:**
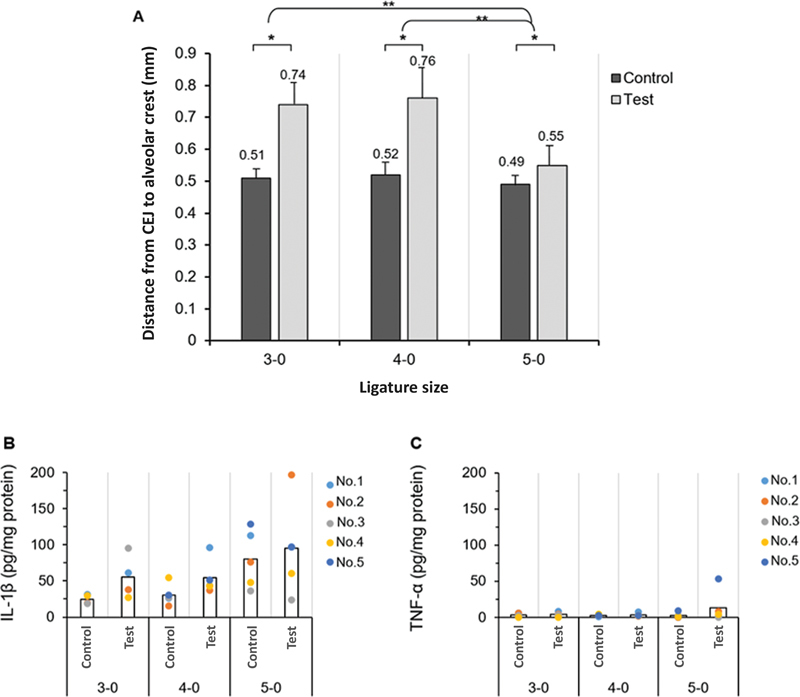
( **A** ) Alveolar bone loss at 2 weeks following placement of ligatures using different ligature sizes. ( *n* = 4-5/group, * *p* < 0.05; Student's *t* -test, ** *p* < 0.05; one-way analysis of variance and Bonferroni's post-hoc tests). ( **B, C** ) Mean proinflammatory cytokine levels of interleukin-1β (IL-1β) and tumor necrosis factor-α (TNF-α) at 2 weeks following placement of ligatures using different ligature sizes ( *n* = 4-5/group, * *p* < 0.05, Wilcoxon signed-rank test). Detection limit for serum IL-1β and TNF-α level was 5 pg/mL. CEJ, cementoenamel junction.

**Fig. 2:**
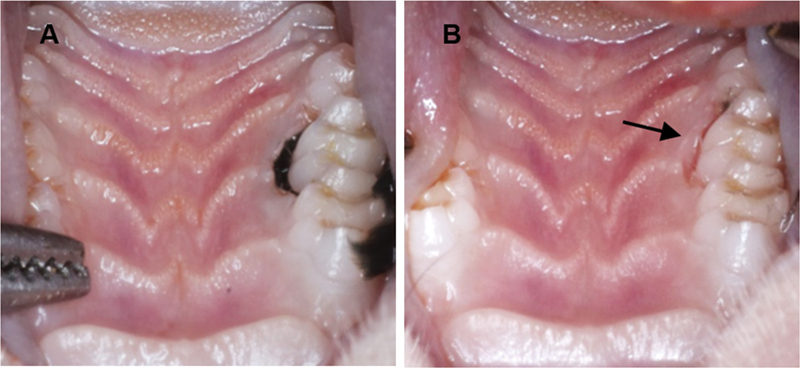
Clinical features of rat gingival tissue. ( **A** ) Maxillary left second molar was ligatured with 3-0 silk for 2 weeks. ( **B** ) Immediately after ligature was removed (day 0). Clinical signs of inflammation were presented (as pointed by arrow).

Alveolar bone loss (the distance between CEJ to ABC) at control sites was not statistically different compared among 3-0, 4-0, and 5-0 groups. At 2 weeks, ligature size 3-0 and 4-0 resulted in significantly greater mean bone loss compared to 5-0 ( *p* < 0.05). Mean alveolar bone loss of ligatured tooth in 3-0, 4-0, and 5-0 groups was 0.74 ± 0.07, 0.76 ± 0.10, and 0.55 ± 0.06 mm, respectively ( Fig. 1A ). There was no statistical difference between ligature size 3-0 and 4-0. In all groups (ligature size 3-0, 4-0, and 5-0), the levels of IL-1β and TNF-α at ligatured tooth were higher than control, although the difference was not statistically significant ( Fig. 1B, C ). There were no statistically significant differences of IL-1β and TNF-α levels among different ligature sizes. These findings were consistent with the clinical manifestations of inflammation including gingival erythema, thickened gingival margin and interdental papilla, and gingival bleeding ( Fig. 2 ).

Therefore, in the following experiment, ligature size 3-0, which contributed to the greatest amount of alveolar bone loss with the lowest percentage of ligature loss, was used.

### Alveolar Bone Healing and Proinflammatory Cytokine Level after Ligature Removal at Different Time Points

**Fig. 3:**
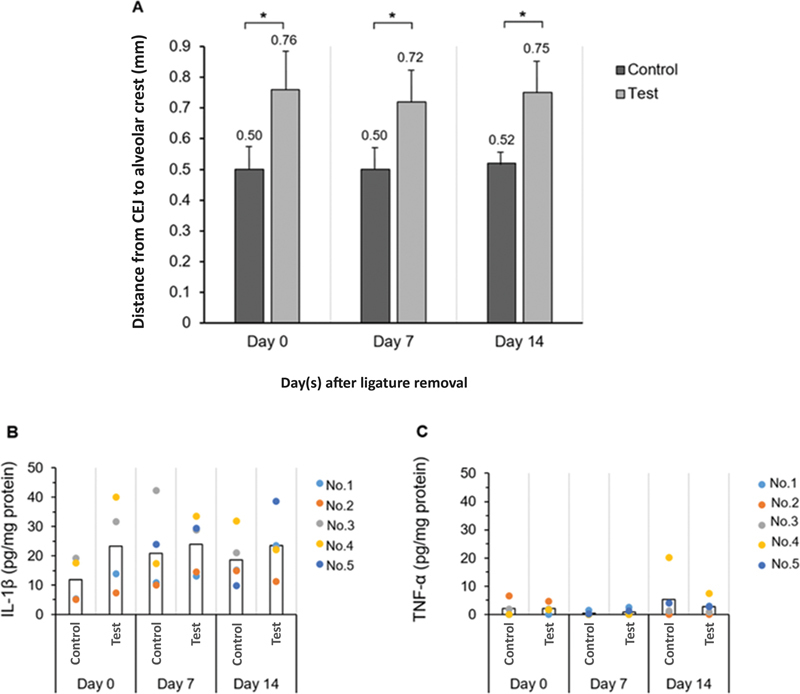
( **A** ) Alveolar bone loss at the indicated days following ligature removal ( *n* = 5-6/group, * *p* < 0.05 compared between test and control, Student's *t* -test). ( **B, C** ) Mean proinflammatory cytokine level at different time points after ligature removal ( *n* = 4-5/group, * *p* < 0.05, Student's *t* -test). Detection limit for serum interleukin-1β (IL-1β) and tumor necrosis factor-α (TNF-α) level was 5 pg/mL. CEJ, cementoenamel junction.

3-0 silk ligature was placed around left maxillary second molar for 2 weeks. After ligature removal, the alveolar bone level was examined at the different time points, including the day of ligature removal (day 0, day 7, and day 14). Alveolar bone loss at the ligatured sites were significantly greater than nonligatured control at all time points ( *p* < 0.05). There was no statistically difference between each time point ( Fig. 3A ).

Alveolar bone healing was calculated by change in alveolar bone level at day 7 or day 14 from day 0. One maxilla from each time point was used for histologic analysis.

Alveolar bone healing at day 7 was 0.05 ± 0.08 mm (buccal 0.07 ± 0.06 mm, palatal 0.02 ± 0.10 mm) and day 14 was 0.03 ± 0.08 mm (buccal 0.09 ± 0.12 mm, palatal −0.02 ± 0.08 mm). There was no statistically significant change between day 7 and 14 after ligature removal.

The level of proinflammatory cytokines at different time points following ligature removal showed no statistically significant differences. The levels of IL-1β and TNF-α were not significantly changed on day 7 and day 14 compared to day 0 ( Fig. 3B, C ).

### Histologic Evaluation

**Fig. 4:**
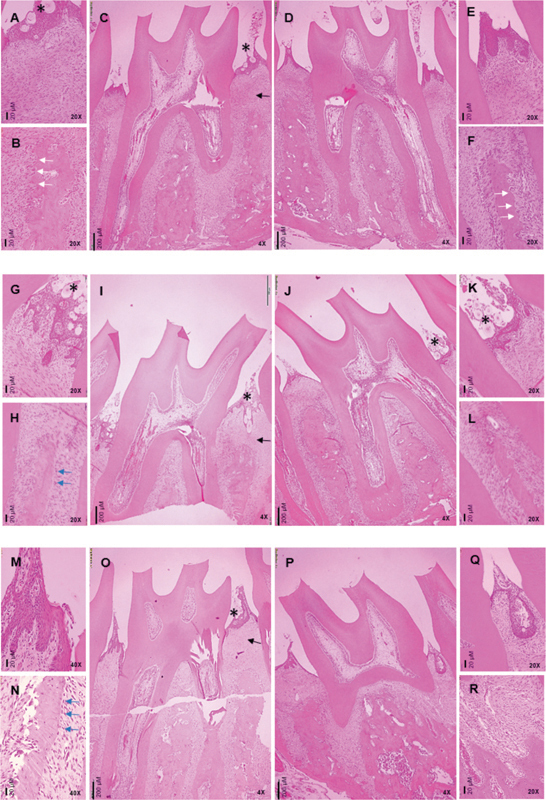
Representative histologic sections of rat maxillary second molar. At day 0, after 2 weeks of ligation by 3-0 silk, the ligatured site ( **A–C** ) exhibited epithelial rupture and foreign bodies ( **A** ), and pronounced surface depressions caused by bone resorption ( **B** ). The control site ( **D–F** ) exhibited intact epithelium ( **E** ), and less surface depressions ( **F** ). On day 7 after removal of ligature, the ligatured site ( **G–I** ) exhibited markedly epithelial ulceration (G), and few osteoblastic-like cells on alveolar bone surface (H), whereas the control site ( **J–L** ) exhibited less epithelial rupture (K). On day 14 after removal of ligature, epithelial rupture ( **M** ) and osteoblastic-like cells on alveolar bone surface (O) were still presented on the ligatured site ( **M–O** ), but not on the control site ( **P– R** ). The black arrows indicate inflammatory cell infiltration, the white arrows indicate bone surface resorption, the blue arrows indicate osteoblastic-like cells, and asterisks indicate foreign bodies.

The amount of attachment and alveolar bone loss corresponded to the results obtained using stereomicroscope and micro-CT. At day 0 (after 2 weeks of ligation), dilated blood vessels and inflammatory cell infiltration such as polymorphonuclear leukocytes, macrophages, and lymphocytes were observed in the subepithelial connective tissue from both ligatured and control sites. Surface resorption of alveolar bone and osteoclasts were observed from both sites, but was more pronounced at the ligatured site ( Fig. 4A–F ). Furthermore, disruption of epithelial integrity and foreign bodies were observed at ligatured site ( Fig. 4A, C ), whereas the epithelium at control site was more intact ( Fig. 4D, E ). The foreign bodies observed may be ligature remnants, plaque, or food debris.

Comparing between the different time-points following ligature removal, inflammatory cell infiltration was evident at day 0 and decreased notably on day 7 ( Fig. 4G–I ) and 14 ( Fig. 4M–O ), respectively. Focusing on the ligatured site, sulcular epithelium on day 7 ( Fig. 4G, I ) was more disrupted than on day 14 ( Fig. 4M, O ). There were some osteoblastic-like cells on the alveolar bone surface at the ligatured site, which were more abundant on day 14 ( Fig. 4N ) compared to day 7 ( Fig. 4H ), but was not found at the control site ( Fig. 4L, R ).

## Discussion

In our study, we found that placing silk ligatures around the maxillary second molar for 2 weeks significantly contributed to alveolar bone loss in rats. While significant bone resorption could be noted after 7 days of ligation, the majority of the increased bone resorption was still observed between 7 and 14 days. Additionally, no statistically significant differences in linear bone loss were observed between 14 and 21 days, suggesting that connective tissue and bone loss occur predictably over a period of 7 to 15 days, and that periodontal disease tends to stabilize during the later periods. 22 Therefore, we chose a 2-week disease induction period. The distance from CEJ to ABC was approximately 0.5 to 0.7 mm. These results were in accordance with the previous studies in rodent which found that the ligature model could rapidly induce alveolar bone loss within 1 to 8 weeks and the distance from CEJ to ABC was about 0.5 to 1.5 mm. 8 9 17 18 19 23 24 Considering the pattern of alveolar bone loss, greater bone loss was observed at the buccal side compared to palatal side. In addition, we noticed that the standard deviation of alveolar bone loss on buccal side was slightly higher than palatal side, which was similar with the earlier study by Abe and Hajishengallis. 8

Several studies have showed that the amount of alveolar bone loss was often correlated with the duration of ligation period, with longer duration causing greater alveolar bone loss. It was demonstrated that alveolar bone loss was increased in the initial period following ligature placement between day 7 and 30, the intensity of bone loss was then decreased, and bone resorption was stabilized over time during day 45 to 60. The authors stated that the decrease in severity of alveolar bone loss over time might be due to the protective features of periodontal tissues that apically migrated away from bacterial plaque violation and to recover the biologic width. 5 25 We observed less alveolar bone loss compared to the previous studies. 8 9 17 18 19 23 24 This could be owing to the shorter ligation period in our study; however, amount of bone resorption occurred was significantly greater than control. The shorter ligation period could lower the cost of the study and shorten the experimental time.

Silk was recommended in ligature-induced periodontitis model as it effectively promoted plaque accumulation. The recommended ligature size, however, was not stated. 11 The comparison of different sizes of ligature in ligature-induced periodontitis in a rodent model has never been investigated. In our study, both 3-0 and 4-0 silk effectively resulted in the alveolar bone loss after 2 weeks of ligation. However, 3-0 silk exhibited a lower rate of ligature loss compared to 4-0 silk. 5-0 silk was less effective in causing alveolar bone loss, and had the highest rate of ligature loss. The rate of ligature loss in our study in 7-week-old rats was 30 to 40% (for silk 3-0 and 4-0), which was higher than Marchesan's study in 8- to 12-week-old mice (<20%). We hypothesized that the use of 8- to 12-week-old rats might be able to reduce the rate of ligature loss in our future studies. Although the rate of ligature loss has never been studied in the rat model, it has been suggested that mice aged between 8 and 12 weeks are suitable for ligature retention. 11 Ligature loss was more prevalent in mice under the age of 8 weeks because younger mice had looser contact between interproximal molars compared to older mice. In contrast, mice older than 12 weeks exhibited tighter contact, resulting in a lower rate of ligature loss. However, this also made it more challenging to place the ligature between the interproximal molars, 11 potentially resulting in periodontal damage during ligature placement.

In this study, we also compared alveolar bone level measurements acquired with a stereomicroscope and a micro-CT. The maxilla was photographed with a stereomicroscope and measured using ImageJ software. 26 For a micro-CT, the maxilla was scanned, and the alveolar bone level was then measured from 3D images reconstruction. We found that alveolar bone level measured from micro-CT images was comparable to data obtained from stereomicroscopic images (data not shown). This showed the validity of both methods.

At the ligatured teeth, there were more plaque accumulation and distinct clinical manifestations of gingival inflammation compared with nonligatured teeth. A higher level of IL-1β was also observed, which correspond to alveolar bone loss on the ligatured teeth. It was implied that the ligature model could promote plaque accumulation around teeth and induce the inflammation and alveolar bone loss, similar to periodontitis occurred in human. However, the mechanism of bone resorption was not confirmed in this study. The difference of IL-1β detected between ligatured and nonligatured teeth was not statistically significant. This could possibly be explained by the timing of tissue collection for cytokine and histological analysis. Our study was conducted on day 14 after ligation, which was later than some other previous studies. 11 25 The amount of TNF-α was very low and could not be detected with the ELISA kit. Thus, TNF-α might not be the best candidate for studying the inflammatory process in this ligature model. In addition to IL-1β and TNF-α, receptor activator of nuclear factor kappa-B ligand (RANKL) also plays a significant role in bone physiology and periodontal bone resorption. 27 28 During the early stages of periodontitis induction in the ligature model (1 and 3 days after ligature implantation), there was a significant increase in the mRNA expression of IL-1β and TNF-α. RANKL mRNA expression, on the other hand, became more prominent in later time periods (3 and 5 days). At 1 week, IL-1β expression remained elevated, but not TNF-α. However, after 14 days, no statistically significant differences were observed for any of the evaluated cytokines. 22 Therefore, it might be of interest to investigate whether RANKL protein levels are elevated in our experimental setting, particularly at the 14-day after ligature placement.

When the ligature was removed from the tooth, the histological inflammation was subsided. However, the alveolar bone level measured was not different compared between day 0, 7 and 14 after ligature removal. This suggested that the spontaneous bone healing did not occur during these 2 weeks period. Thus, it might be interesting to follow up longer. If the bone healing does not occur, this ligature-induced alveolar bone loss can be so-called critical size defect.

Bone healing after removal of ligature was observed from previous studies in ligature-induced periodontitis models. Previous study reported that the amount of alveolar bone loss (distance from CEJ to ABC) was slightly decreased at 2 weeks after the ligature was removed compared to 1-week postligation. It was suggested that, without the ligature, inflammation was resolved and bone healing process could occur. 9 In ligature-induced periodontitis model in diabetic rats, the number of apoptotic fibroblasts was increased, and new bone formation after 4 to 9 days of ligature removal was increased. 7

Histologically, ligated teeth exhibited inflammatory infiltration in the gingival epithelium and connective tissue, which was similar to periodontitis in human. After the ligature was removed, the intensity of inflammatory cell infiltration decreased from day 7 to day 14. The presence of epithelialization and some osteoblastic-like cells on the bone surface at day 14 suggested that the healing process had taken place.

According to the findings of this study, a ligature-induced periodontitis model in rat using 3-0 silk ligated around maxillary second molar for 2 weeks effectively promotes plaque accumulation, gingival inflammation, and alveolar bone loss, similar to periodontitis in human. After ligature removal, the decreased inflammatory level of gingival tissue was observed histologically. Nevertheless, only limited bone healing could be observed during 2 weeks period after the ligature was removed. Therefore, this 2-week period could be used as a window period for testing the regenerative materials and/or treatment modalities in ligature-induced periodontitis model in rat.

## Conclusion

A silk ligature significantly induced alveolar bone loss at ligated teeth in a rat model of experimental periodontitis. Compared to 4-0 and 5-0 silk, 3-0 silk is the most effective ligature size in causing the alveolar bone loss, with the lowest rate of ligature loss. Furthermore, spontaneous bone healing was not observed throughout the 2-week period following removal of the ligature.
